# Tunable 3D Nanofiber Architecture of Polycaprolactone by Divergence Electrospinning for Potential Tissue Engineering Applications

**DOI:** 10.1007/s40820-018-0226-0

**Published:** 2018-10-25

**Authors:** George Z. Tan, Yingge Zhou

**Affiliations:** Department of Industrial, Manufacturing and Systems Engineering, Texas Tech University, Lubbock, TX USA

**Keywords:** Divergence electrospinning, 3D nanofiber scaffold, Tissue engineering, Microstructure gradient

## Abstract

The creation of biomimetic cell environments with micro and nanoscale topographical features resembling native tissues is critical for tissue engineering. To address this challenge, this study focuses on an innovative electrospinning strategy that adopts a symmetrically divergent electric field to induce rapid self-assembly of aligned polycaprolactone (PCL) nanofibers into a centimeter-scale architecture between separately grounded bevels. The 3D microstructures of the nanofiber scaffolds were characterized through a series of sectioning in both vertical and horizontal directions. PCL/collagen (type I) nanofiber scaffolds with different density gradients were incorporated in sodium alginate hydrogels and subjected to elemental analysis. Human fibroblasts were seeded onto the scaffolds and cultured for 7 days. Our studies showed that the inclination angle of the collector had significant effects on nanofiber attributes, including the mean diameter, density gradient, and alignment gradient. The fiber density and alignment at the peripheral area of the 45°-collector decreased by 21% and 55%, respectively, along the *z*-axis, while those of the 60°-collector decreased by 71% and 60%, respectively. By altering the geometry of the conductive areas on the collecting bevels, polyhedral and cylindrical scaffolds composed of aligned fibers were directly fabricated. By using a four-bevel collector, the nanofibers formed a matrix of microgrids with a density of 11%. The gradient of nitrogen-to-carbon ratio in the scaffold-incorporated hydrogel was consistent with the nanofiber density gradient. The scaffolds provided biophysical stimuli to facilitate cell adhesion, proliferation, and morphogenesis in 3D.
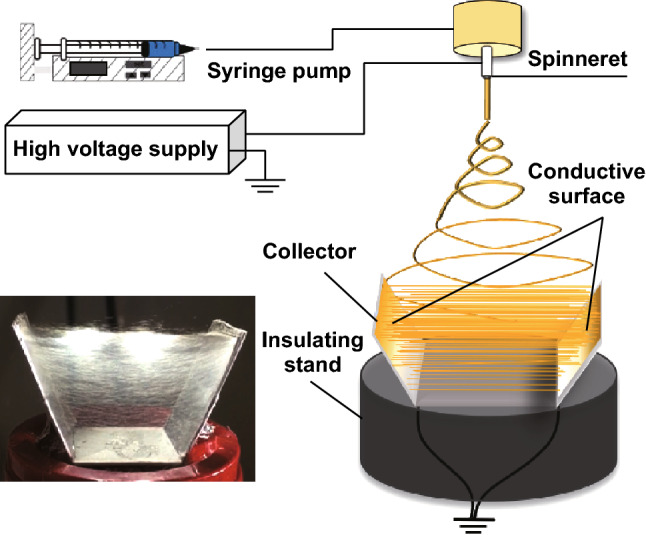

## Highlights

- A novel 3D divergence electrospinning technique of tunable fibrous microarchitecture for tissue engineering.
- Versatile capability of controlling both the microstructure and macroscopic shape of the scaffold.
- Nanofiber scaffold with microstructure gradient coupled with element gradient.

## Introduction

The fabrication of biomimetic cell microenvironments closely resembling native tissues is critical for regenerative medicine. Recent bioinspired approaches have focused on creating biomimetic cell microenvironments that closely resemble the natural gradients of cell distribution, extracellular matrix (ECM), and tissue topology. Studies have shown that micro and nanotopography and the local environment of the ECM influence trends in cell behavior by providing biochemical and biophysical stimuli to promote cell adhesion, proliferation, morphogenesis, and motility [1, 2]. Critical physical features of the ECM include dimensionality, architecture, stiffness, ligand topography, and density [3]. One of the important biofabrication strategies is to integrate tunable microarchitecture in heterogeneous scaffolds to closely resemble the patterned structures of native tissues [4, 5].

Electrospinning has been extensively studied as a nanofiber fabrication technique for tissue engineering. Two-dimensional (2D) mats composed of aligned or grid nanofibers can be created by adopting rotation collectors or arrayed pins. Some important electrospinning-based methods to construct three-dimensional (3D) nanofiber structures include vertically stacking layers of nanofiber membranes [6], incorporating nanofibers in hydrogels [7], rolling nanofiber mats into a tubular structure [8], and combining nanofibers with 3D printing [9]. In addition, a 3D microfiber architecture can be fabricated through melt electrospinning [10]. Our research group discovered that with tailored electrical and rheological properties of polymer solutions, a divergent electric field induced a fiber-bridging phenomenon between two separately grounded bevels, resulting in self-assembly of patterned nanofiber arrays into a functional architecture. In this study, we present a novel 3D divergence electrospinning technique of preparing tunable fibrous microarchitecture for potential musculoskeletal tissue engineering. Divergence electrospinning could control not only the microstructure of the highly aligned nanofiber scaffold, but also the macroscopic shape of the scaffold. In addition, hydrogels with element gradients were fabricated by incorporating the element-loaded nanofiber scaffolds. The scaffolds provided microtopographical cues to promote cell adhesion, proliferation, and morphogenesis. This approach enables the integration of 3D microtopographical cues and biomolecular gradients in a one-station top-down additive manufacturing process.

## Experimental Methods

### Configuration of the Divergence Electrospinning

**Fig. 1:**
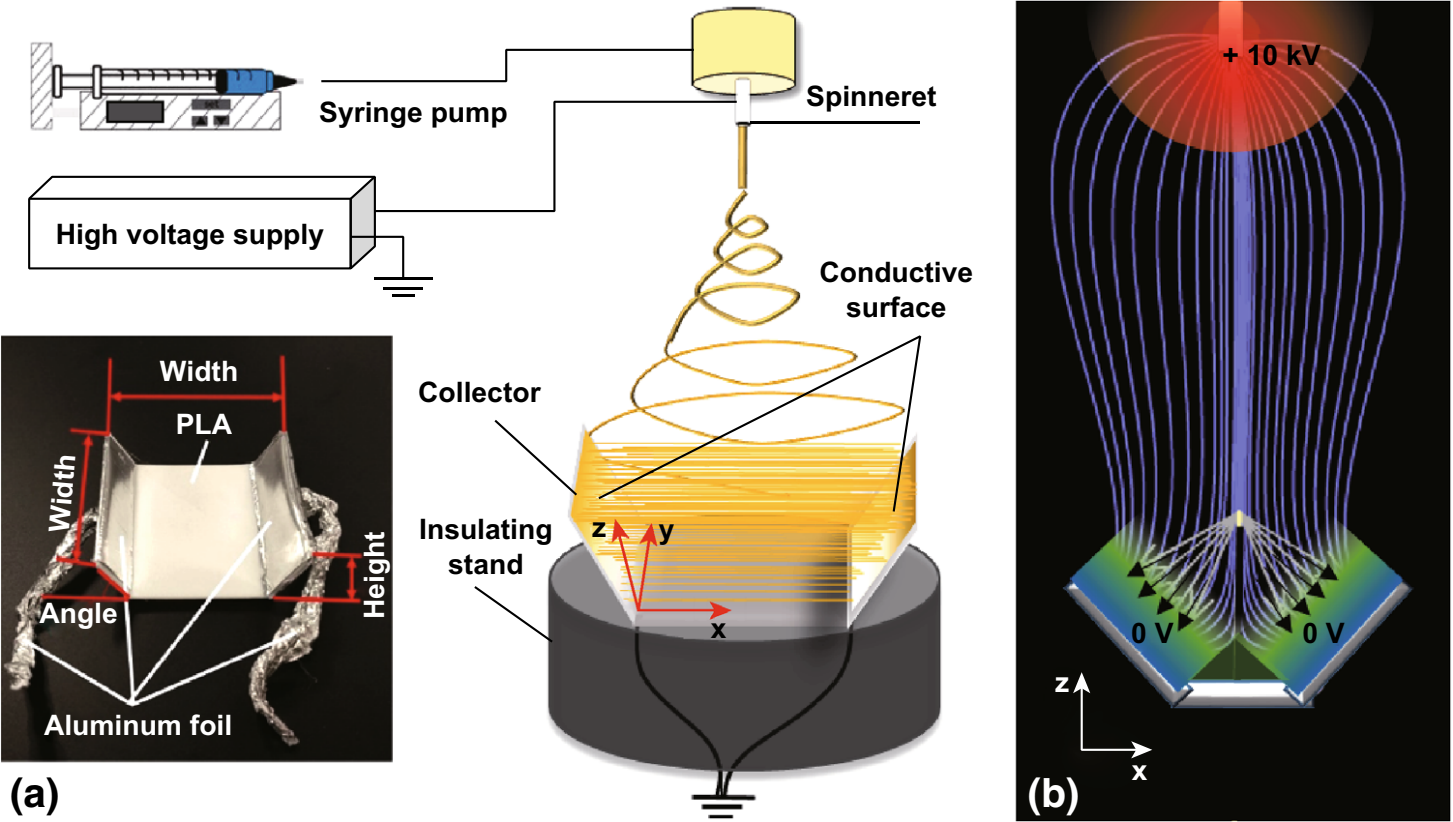
**a** Divergence electrospinning configuration. **b** The divergent electric field between the spinneret and the collector

The divergence electrospinning system was composed of three modules: a high-voltage direct current (DC) source, a single-spinneret solution feeding unit, and a composite double-bevel collector (Fig. 1). The substrate of the double-bevel collector was made of polycaprolactone (PCL) and 3D-printed by fused deposition modeling (Ultimaker^®^ 3, the Netherlands). The inner surfaces of the two axisymmetric bevels were coated with aluminum foil, which was grounded by connecting wires passing through the collector base and an insulating stand. The spinneret was placed above the central line of the collector.

We adopted 15% (w/v) PCL solution as the nanofiber material. The solution was prepared by dissolving PCL pellets (MW = 80,000) in N,N-dimethylformamide and chloroform (1:1) through magnetic stirring for 4 h at room temperature. All materials above were purchased from Sigma-Aldrich^®^ (St. Louis, MO). To stain the nanofibers, 0.1 mg mL^−1^ DilC_12_(3) perchlorate (Thermo Fisher Scientific, Waltham, MA) was added to the PCL solution. We first adopted a preset collector (width = 40 mm, height = 40 mm, inclination angle = 60°) to determine the appropriate ranges of process parameters. Through the preliminary study, the electric field intensity and the pump rate were set to be 1.1 kV cm^−1^ and 0.375 mL h^−1^, respectively. Then we investigated the effects of inclination angle on the nanofiber attributes. Two levels of angles, 45° and 60°, were tested. The width and height of the scaffolds were 40 and 20 mm, respectively.

### Characterization of Scaffold Microstructure

The 3D microstructures of the nanofiber scaffolds were characterized through a series of sectioning in both vertical and horizontal directions. Vertical sectioning was performed at four equidistant points across the y-axis (Fig. 1a) of the scaffold using thin glass slides attached with double-sided tapes. The distance was 5 mm for the 20 mm-wide collectors and 10 mm for the 40 mm-wide collectors. Horizontal sectioning was performed at seven equidistant points (distance = 1.1 mm) across the *z*-axis. The sectioned samples were observed by scanning electron microscopy (SEM, Phenom ProX, NanoScience, Alexandria, VA) and analyzed using ImageJ. The images were segmented and processed for measurement of fiber diameter, fiber density, and fiber alignment.

### Changes in Scaffold Shape

Given that the nanofibers were only deposited on the grounded area, we hypothesized that the scaffold shape could be altered according to the profiles of the conductive areas on the collector bevels. To test this hypothesis, we adopted four different geometries of conductive areas: a triangle with vertical orientation, a triangle with horizontal orientation, a circle, and a circle with a concentric hole. In addition, a four-bevel collector (with a rectangular conductive area) was adopted to test whether a 3D nanofiber scaffold with a grid structure could be obtained.

### Hydrogel with Element Gradient

To demonstrate the potential of incorporating element gradients by divergence electrospinning, we fabricated PCL/collagen nanofiber scaffolds through the basic two-bevel configuration. The solution was prepared by mixing 10% (w/v) collagen type I powder and 10% (w/v) PCL in hexafluoroisopropanol. The electrospun scaffolds were placed in 2% sodium alginate solution and cross-linked by 2% CaCl_2_ solution. The nanofiber-incorporated hydrogel was freeze-dried at − 50 °C for 48 h. The cross-sections of the dried hydrogel scaffolds were examined by energy-dispersive X-ray spectroscopy (EDS) for elemental composition analysis. We selected nine equally spaced spots along the *z*-axis of the scaffold for EDS and calculated the nitrogen-to-carbon (N/C) ratio at each spot. The fitting curve for each scaffold was generated based on the nine points, and the gradient of N/C ratio was plotted by MATLAB.

### Cell Culture

We performed a cell culture study to investigate the potential effect of the biomimetic nanofiber architecture on cell growth. Human fibroblasts (ATCC^®^ MRC-5) were seeded on UV-sterilized electrospun scaffolds at a concentration of 2 × 10^5^ cells mL^−1^ in Eagle’s Minimum Essential Medium (ATCC^®^, Manassas, VA) with 10% fetal bovine serum (ATCC^®^, Manassas, VA). The scaffold was incubated at 37 °C (CO_2_ = 5%) for 1 day for cell attachment. Because some cells were attached to the bottom of the well instead of the nanofiber scaffold, the scaffold with cells was transferred to another well and cultured continuously for 7 days. Alamar Blue (Thermo Fisher Scientific, Waltham, MA) tests were conducted on day 1 and 7 to quantify cell proliferation. After day 7, cells were fixed with 4% formaldehyde and stained with Phalloidin CruzFluor™ 488 Conjugate (Santa Cruz Biotechnology, Dallas, TX) and 4′,6-diamidino-2-phenylindole (Santa Cruz Biotechnology, Dallas, TX) for filamentous F-actin and nuclei, respectively. Fluorescence images were taken from both the top and side of the scaffold.

## Results and Discussion

**Fig. 2:**
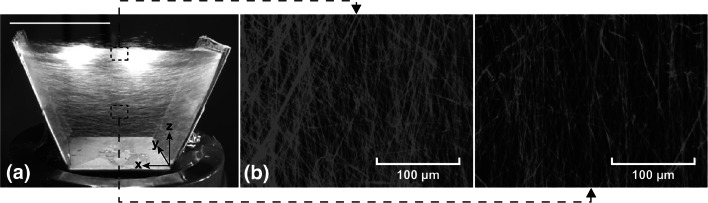
**a** 3D nanofiber scaffold obtained by the double-bevel collector. Scale bar represents 20 mm. Fluorescent nanofibers at the **b** top section and **c** bottom section of the scaffold

When a high-voltage DC was applied to the spinneret, the double-bevel collector induced a divergent electric field, which resulted in a deposition of nanofibers onto the inner surfaces of both bevels and simultaneously led to the self-assembly of aligned nanofibers between the two bevels. After 2 min of electrospinning, uniaxially aligned nanofibers were accumulated between the two grounded bevels, creating a bundle-form scaffold with high porosity (Fig. 2). The thickness of the nanofiber scaffold was about 2 cm. The porosity within the scaffold increased from the top (41.3%) to the bottom (85.4%).

**Fig. 3:**
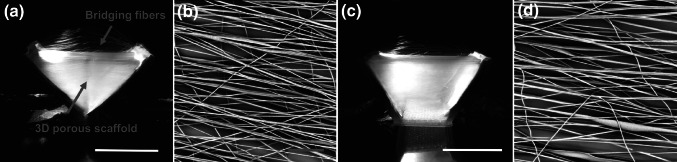
3D nanofiber scaffolds formed in the **a** 45°-collector and **c** 60°-collector. SEM pictures of the nanofibers at the **b** top of the 45°-collector and **d** 60°-collector. White scale bar = 10 mm and red scale bar = 10 µm. (Color figure online)

**Fig. 4:**
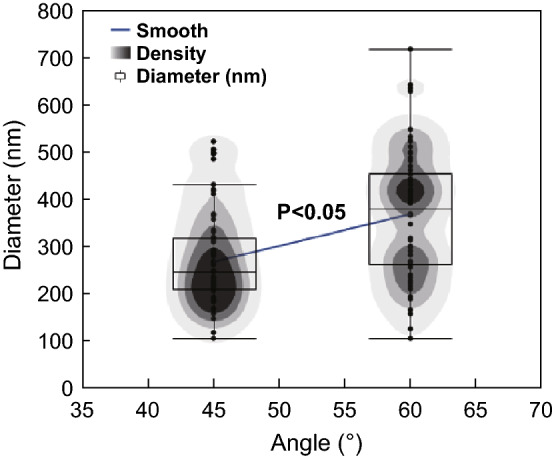
Violin boxplots for nanofiber diameter (nm) distribution in the two collectors

The scaffolds formed in the two collectors with different inclination angles are shown in Fig. 3. Both scaffolds were composed of aligned nanofibers. As shown in Fig. 4, the diameter of the nanofibers ranged from 100 to 730 nm. The *t* test showed that there was a significant difference (*p* < 0.05) between the diameters of the two scaffolds. The mean diameter of the nanofibers in the 60°-collector was approximately 48% larger than that in the 45°-collector.

**Fig. 5:**
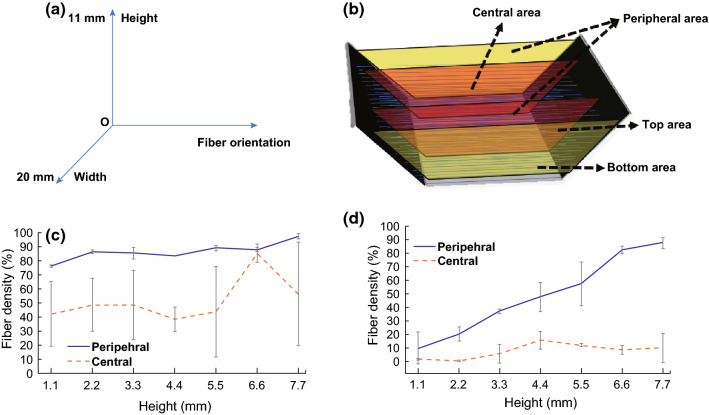
**a** The coordinate system of the scaffold. **b** An illustration of different areas of a scaffold. The width of the two peripheral areas is 5 cm. The width of the central area is 10 cm. Fiber density gradients in the **c** 45°-collector and **d** 60°-collector

The fiber densities of the two scaffolds are summarized in Fig. 5. For both collectors, the fiber density was higher in the peripheral and top area than in the central and bottom area. The fiber density at the peripheral area of the 45°-collector was consistently high along the vertical direction (*z*-axis), while that of the 60°-collector decreased by 71% from the top to the bottom. Similarly, the fiber density at the central area of the 45°-collector varied from 25 to 90%, but that of the 60°-collector remained below 20%. Collectively, the 45°-collector resulted in a more homogeneous fiber distribution along the vertical direction, while the 60°-collector caused a substantial increasing trend from the bottom to the top. This phenomenon might explain the difference between the mean fiber diameters of the two scaffolds. The nanofiber diameter was negatively correlated to the jet travel distance (spinneret-to-ground distance) and thus, more fibers at the bottom of the collector would result in a lower mean value.

**Fig. 6:**
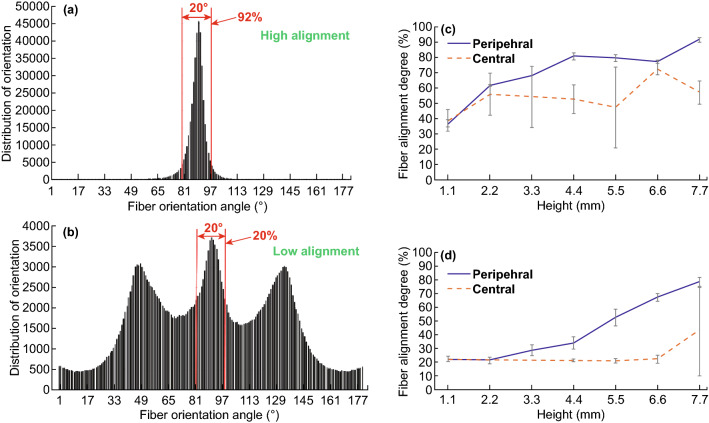
**a** A histogram of fiber orientation at 7.7 mm from the bottom of the peripheral area in the 45°-collector. **b** A histogram of fiber orientation at 4.4 mm from the bottom of the central area in the 60°-collector. Fiber alignment gradients in the **c** 45°-collector and **d** 60°-collector

The fiber alignment degree is defined as the percentage of fibers within ± 10° of the peak angle. Examples of high alignment and low alignment are presented in Fig. 6a, b, respectively. In Fig. 6a, 92% of fibers were within ± 10° of the peak angle, while in Fig. 6b, only 20% of fibers were within this range. The gradient of fiber alignment was also influenced by the inclination angle. As shown in Fig. 6c, d, there was a higher consistency in fiber alignment across the *z*-axis of the scaffold in the 45°-collector compared to that in the 60°-collector. The overall degree of fiber alignment was higher in the peripheral and top area than in the central and bottom area. In our pilot study, an inclination angle of 90° only yielded a 2D fiber mat on the top of the collector. Therefore, tilted bevels (inclination angle < 90°) are critical to form 3D nanofiber scaffolds. When the angle decreases from 60° to 45°, the electric field becomes more divergent and thus induces a higher homogeneity in fiber density. The fibers at the bottom tend to be less aligned than those at the top largely because of the instability at the early phase of electrospinning. Thus, the fiber alignment gradients tend to be consistent with the fiber density gradient. We believe that by controlling the fiber density gradient, nanofiber scaffolds can be applied to engineering of both homogenous [11] and heterogeneous [6] tissues. PCL is a biodegradable and thermoplastic polymer possessing high mechanical properties. Such features are essential for the development of scaffolds for musculoskeletal tissues. During divergence electrospinning, we observed that the deposited nanofibers were slightly pressed downward by the impact of jet whipping, causing a secondary elongation that does not exist in 2D electrospinning. It is not clear how 3D assembly affects the crystalline structures and mechanical properties of individual nanofibers compared to 2D electrospinning processes. Future studies will include crystallization characterization and nanomechanical tests of the electrospun fibers, such as assessing the bending Young’s moduli by atomic force microscopy and quantitative nanomechanical mapping [12].

**Fig. 7:**
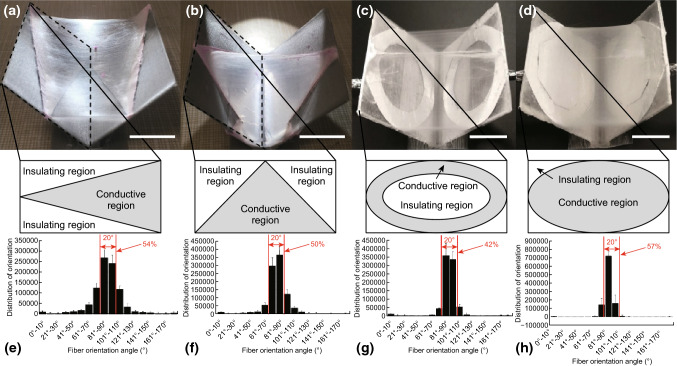
**a** A pentahedral and **b** tetrahedral scaffold fabricated by specially designed collectors. **c** A columnar scaffold with a hollow structure and **d** a cylindrical scaffold. Scale bar = 10 mm. **e**–**h** The fiber alignment histograms for scaffolds 7a–7d, respectively

It was found that the conductive areas at the collector bevels had a deterministic effect on the 3D geometry of the electrospun scaffold. As shown in Fig. 7a, b, a pentahedral and a tetrahedral scaffold were created by adopting triangular conductive regions because the nanofibers assembled solely on the conductive region. These two designs showed that polygon-shaped scaffolds could be directly electrospun based on the linear changes in the conductive areas in both horizontal and vertical directions. Similarly, in Fig. 7c, d, an asymmetric cylindrical scaffold and a columnar scaffold with a hollow structure were fabricated by adopting elliptical and cavity conductive areas, respectively. It was found that all four collectors resulted in highly aligned nanofiber structures. The collector with an elliptical conductive area (Fig. 7h) caused the highest degree of alignment, while the collector with a horizontal triangle area (Fig. 7e) caused the lowest degree of alignment. This phenomenon has important implications in biofabrication and other microfiber engineering areas because for the first time, a 3D nanofiber scaffold with tunable geometry was directly fabricated through electrospinning. Divergence electrospinning showed a versatile capability to control not only the microstructure of the nanofiber scaffold, but also the macroscopic shape of the scaffold.

**Fig. 8:**
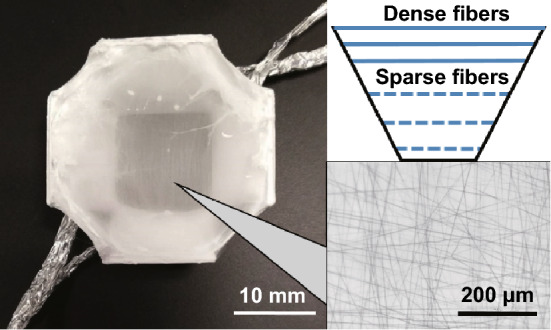
A mesh-structure scaffold fabricated by the four-bevel collector

Using a four-bevel collector, a 20 mm × 20 mm scaffold with a grid microstructure was obtained as shown in Fig. 8. Multi-axially aligned nanofibers assembled at the top section of the collector. Fibers connecting each pair of bevels formed a matrix of microgrids with a density of ~ 11%. The volumetric density of reticular fibers varies from 10 to 30% depending on the locations of the tissues as well as the age of person [13]. The special internal structure resembles reticular fibers in connective tissues and has the potential to facilitate large-scale cell proliferation because of the large space for cell adhesion. It was noted that the thickness of this grid structure was only 1.3 mm, which is much lower than that of scaffolds formed in the two-bevel collectors. Sparse nanofibers with different orientations were distributed at the lower section of the collector, but there was no grid structure. We speculate that the four-bevel collector further dispersed the electric field and reduced the energy density on each bevel and thus, fewer fibers were deposited at the lower section of the collector. A mechanism-based model will be developed in the future to validate this assumption and to optimize the process parameters for controlling the thickness of the nanofiber grid structure.

**Fig. 9:**
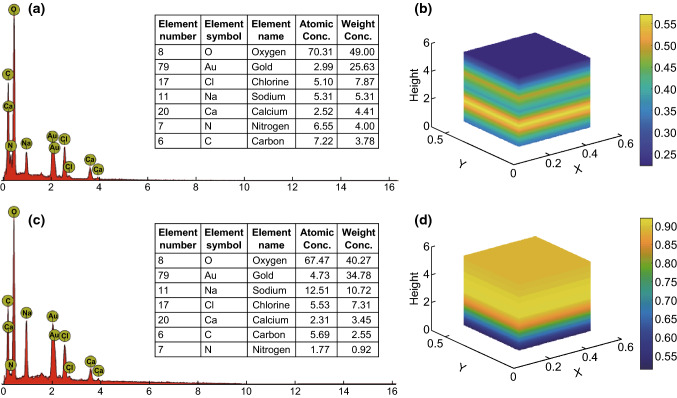
**a** EDS and spectrum for top region in collector, **b** Element gradient plot for collector, **c** EDS and spectrum for top region in collector, **d** Element gradient plot for collector (Collectors: width = 20 mm, height = 10 mm, angle = 45°). Color bar represents the ratio of nitrogen to carbon. (Color figure online)

In addition to microstructure gradient, element gradient in scaffolds is also important for tissue engineering and has been extensively studied as a delivery method for drugs and growth factors [14, 15]. For the hydrogel incorporated with PCL/collagen nanofiber scaffold, the N/C ratio along the *z*-axis was plotted by MATLAB (Fig. 9). An obvious element (nitrogen) gradient was observed in both samples. Consistent with our hypothesis, the element gradient was highly correlated to the fiber density gradient. This result showed the potential of using nanofiber scaffolds as a delivery vehicle to create a heterogeneous environment with gradients of elements. The element gradient is important for tunable non-homogenous drug/growth factor release [16, 17], leading to various applications for bone [18], skin [19], neural [20], and vessel tissue vascularization. For example, scaffolds with growth factor gradients were used to accelerate vascularized tissue formation in vivo [21]. Scaffolds with bioactive molecule concentration gradients were found to orient and increase the growth of rat dorsal root ganglion neurons compared to a single uniform bioactive molecule concentration [22].

**Fig. 10:**
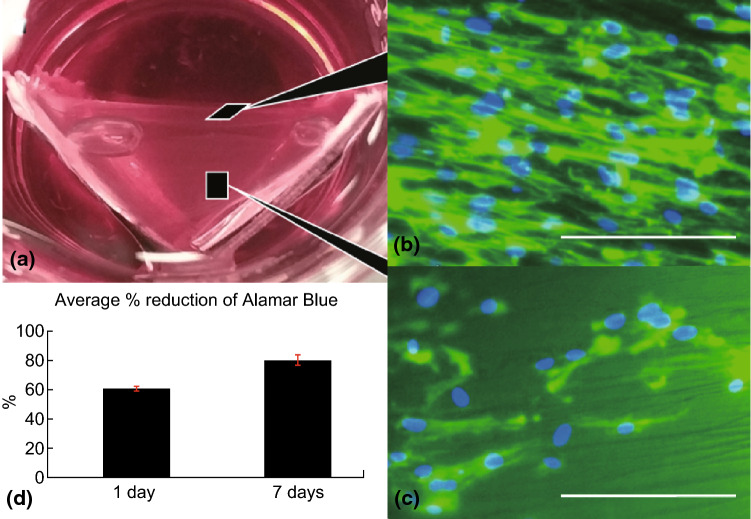
**a** A cell-seeded nanofiber scaffold in culture medium. Fluorescence images of fibroblasts from the **b** top and **c** side view of the scaffold. Scale bar = 200 μm. Blue: nucleus; green: cytoplasm. **d** Average percentage reduction of alamar Blue dye after day 1 and 7. (Color figure online)

The stained cells on the nanofiber scaffold are presented in Fig. 10. The cytoskeletons were stretched along the nanofiber direction, and the cells were distributed in 3D space. The organization of the cells closely resembled the native musculoskeletal tissue structure. The reduction of alamar Blue increased by over 20% (*p* < 0.05), indicating a substantial increase in viable cells over the 1-week period. This result is consistent with many previous studies [23, 24] on aligned nanofiber scaffolds, in which nanofibers provided contact guidance to migration and extension, resulting in an elongation and alignment of the cytoskeleton and nucleus along the axes of the fibers [25]. The patterned fibrous microstructure is considered to optimize the material’s response to external loading [26] and enable specific cell migration during tissue regeneration [27, 28]. It is critical for regeneration of cartilage and musculoskeletal tissues, including tendon and ligament. In addition, the electrospun scaffold retained its shape since the aligned nanofiber array provided necessary mechanical strength to keep its structural integrity. Therefore, there is no need to construct a 3D scaffold by post-processing 2D mats. Once fabricated, the cell-seeded nanofiber scaffold can be directly incorporated into hydrogels, such as sodium alginate, with intact microarchitecture.

While 2D scaffolds have been widely used in tissue engineering applications, they fall short in investigating the factors proven to be crucial to an in vivo environment, such as cell communication in the context of ECM, mechanical cues, and nutrient transportation [29]. Although tremendous effects have been made in creating 3D nanofiber scaffolds, including post-processing nanofiber mats [30–32], self-bundling of random nanofibers with selective polymers or conditions, and adopting ground-pin arrays [33], solution-based electrospinning is still widely considered as a 2D or 2.5D manufacturing process [34]. Moreover, electrospun nanofiber mats tend to have a high fiber density, which impedes cell infiltration. Divergence electrospinning overcomes the drawbacks and enables the rapid assembly of uniaxially aligned nanofibers into centimeter-scale 3D scaffolds with a tunable gradient in fiber density. It is not clear what causes the fiber density gradient along the *z*-axis of the scaffold. We speculate that the electrostatic repulsion due to the residual charges on the nanofibers result in the low density at the bottom of the scaffold. Thus, the homogeneity of nanofiber distribution can be enhanced by dissipating charges on the deposited fibers. A potential strategy will be adjusting the conductivity of the nanofibers by introducing conductive polymers or salts in polymer solutions.

## Conclusion

A novel 3D method of polymer microfiber array assembly was explored in this study as a one-station, top-down approach for integration of biomimetic microarchitecture with tissue scaffold. Our study showed the feasibility of direct electrospinning of centimeter-scale scaffolds with tunable nanofibrous structures within several minutes. The inclination angle of the collector influenced the nanofiber attributes, including diameter, density, and alignment. By altering the projection geometry on the collecting bevels, a polyhedron and a cylinder composed of aligned nanofibers were directly fabricated through divergence electrospinning. A grid nanofiber structure was also created by adopting a four-bevel configuration. In addition, hydrogels with element gradients were made by incorporating the element-loaded nanofiber scaffolds. The scaffolds provided microtopographical cues to promote cell adhesion, proliferation, and morphogenesis in 3D. In conclusion, divergence electrospinning provides a highly efficient and scalable biofabrication platform for nanofiber scaffolds with both microstructure and element gradients. This technique will promote the development of novel nanoarchitecture with modulated functionality, composite materials, and complex features in response to the dynamic physiological and mechanical environments for biomedical application. It will facilitate the development of biomimetic artificial tissues with patterned nanofiber structures, such as tendon, ligament, cartilage, and muscle.
